# What drives emotional escalation in crisis talk? Collectivenism, topics, and LLM-assisted measurement on Zhihu

**DOI:** 10.3389/fpsyg.2026.1724075

**Published:** 2026-04-15

**Authors:** Yu Su, Tongtong Li

**Affiliations:** 1 School of Journalism and Communication, TsingHua University, Beijing, China; 2 School of Journalism, Fudan University, Shanghai, China

**Keywords:** collectivism, cultural values, emotional intensity, individualism, social media discourse

## Abstract

**Introduction:**

This study investigates the emotional responses expressed on Zhihu during the static management period of Shanghai’s 2022 lockdown, focusing on the influence of cultural values, specifically individualism and collectivism, on the intensity of negative emotion.

**Methods:**

Using a combination of GPT-3.5 sentiment intensity labeling, a value orientation lexicon, and *K*-means topic clustering, we analyzed 3,881 posts related to the COVID-19 pandemic.

**Results:**

The results reveal that overall, discussions were highly negative, with collectivist-oriented texts expressing stronger negative emotion than individualist-oriented texts, which were more neutral or positive. Additionally, we found that topics related to medical resources and COVID-19 policy implementation intensified negative emotion. The “daily life and work” topic emerged as a key moderator, further amplifying emotional differences between collectivism and individualism, with collectivism displaying more pronounced negative emotion and individualism showing more moderate emotional responses.

**Discussion:**

This research fills a gap in the existing literature by integrating individualism–collectivism into emotional intensity studies and highlights the potential for differentiated communication strategies by governments and platforms, particularly in addressing high-risk topics and engaging culturally diverse value groups.

## Introduction

The COVID-19 pandemic triggered profound global emotional responses through stringent lockdowns. In China, the “dynamic zero-COVID” strategy created significant social isolation (Xinhua News, 2022), exemplified by the 2022 Shanghai lockdown—officially termed “static management”—which restricted movement for over 2 months (Cao, 2022; The Paper, 2022; Zhou and Zhu, 2022). During this period, Shanghai residents were subjected to substantial negative emotional strain (Zhang et al., 2022).

This surge in negative affect was closely linked to the chronic stress induced by the prolonged pandemic. Research indicates that repeated lockdown measures significantly exacerbate individual anxiety and worry, leading to a continuous deterioration in mental health (Dhensa-Kahlon et al., 2025; Shekriladze et al., 2022). By 2022, the third year of strict pandemic protocols in China, the public was experiencing deep fatigue. Coupled with an outbreak scale that surpassed the initial 2020 Wuhan epidemic (Liu and Du, 2022), negative emotions inevitably became both pervasive and intense.

Existing research often explores emotional responses during public health crises through the lens of cultural values, typically finding that collectivists comply more readily and experience less negative affect than individualists (e.g., Ahuja et al., 2021; Gong et al., 2022). However, these studies have predominantly focused on the early stages of the pandemic; the relationship between cultural values and emotional responses remains under-explored in the context of the late-pandemic era, particularly under extreme containment measures. Simultaneously, the emotional fluctuations triggered by frequent lockdowns prompted this study to move beyond mere affect prevalence and to focus specifically on emotional intensity. In light of this, using the 2022 Shanghai lockdown as an extreme late-pandemic case (Zhao et al., 2024), this research explores how the intensity of negative emotional responses is influenced by divergent cultural value orientations.

By analyzing 3,881 posts from Zhihu, a prominent Chinese Q&A platform where a predominantly highly educated user base engages in analytical, long-form discourse that offers significantly greater depth than microblogging services (Zhang, 2020; Ru and Hu, 2016), through a tripartite approach, incorporating GPT-3.5 sentiment labeling, a value orientation lexicon, and *K*-means clustering, this study found that cultural value orientations significantly influenced emotional intensity. Notably, collectivist-oriented discourse expressed negative emotions with greater intensity than individualist-oriented content, particularly regarding daily life topics, which acted as a moderator amplifying this emotional disparity.

The significance of this study is twofold. Theoretically, it incorporates emotional intensity into the individualism–collectivism framework, filling a gap regarding intensity variations under chronic persistent stress in a late-pandemic context. Practically, it provides a roadmap for government agencies and platforms to adopt culturally tailored communication strategies, ultimately enhancing the efficacy of public health messaging during large-scale crises.

## Cultural values and public health crisis

Collectivism, defined by the prioritization of group cohesion and collective goals over individual autonomy (Hofstede, 1980, 2011; Schwartz, 1990), typically manifests through interdependent relationships within hierarchical societies that emphasize in-group solidarity and respect for authority. Individualism, conversely, rests on the premise of individual independence (Oyserman et al., 2002) and emphasizes the primacy of personal identity, rights, and needs over those of the collective (Ting-Toomey and Dorjee, 2018).

Consequently, individuals oriented towards collectivism tend to define themselves through their social groups, prioritize collective goals, and demonstrate strong adherence to collective norms and responsibilities (Triandis, 2001). In contrast, individuals oriented towards individualism place greater value on personal freedom (Wei et al., 2024) and focus primarily on personal gains and losses (Li et al., 2019).

These concepts function as fundamental cultural values that influence diverse aspects of life, ranging from social structures to individual preferences, attitudes, and perceptions (Grossman and Varnum, 2015; Maravi et al., 2021; Oyserman et al., 2002; Rajkumar, 2021; Triandis, 1995). These values exhibit distinct geographical variations. For instance, populations in Western Europe and North America generally display a pronounced individualistic orientation (Bond and Smith, 1996; Schulz et al., 2019; Talhelm et al., 2014), whereas most East Asian nations lean towards a collectivist perspective (Markus and Kitayama, 2010). Historically, Chinese cultural traditions emphasize the integration of the individual within the collective, stressing the subordination of individual interests to group welfare; in conflicts of interest, the needs of the individual or their immediate group are expected to yield to those of the larger collective (Yang and Xu, 2021).

This distinction becomes particularly salient during public health crises such as COVID-19, especially concerning compliance with preventive measures. Wei et al. (2024) observed that in collectivist societies like China, individuals demonstrated greater willingness to comply with mandatory preventive measures, often driven by beliefs in collective efficacy. Conversely, in individualistic societies like the United States, compliance correlated more strongly with personal self-efficacy. Corroborating this, Lu J. G. et al. (2021) utilized extensive international data to demonstrate significantly higher rates of mask-wearing in collectivist regions compared to individualist ones, even after controlling for other social and demographic variables. Similarly, Cho et al. (2022) found that collectivism was negatively correlated with COVID-19 transmission rates and positively associated with the adoption of preventive behaviors, including vaccination, attributing this partly to the social pressure felt by collectivist individuals to act for the common good.

Cultural value orientations therefore provide a crucial psychological framework for understanding responses to public health measures. When confronted with collective threats like COVID-19, collectivism tends to enhance prosocial behaviors, including active cooperation with government directives, self-regulation, and coordinated collective action (Chen and Peng, 2021). This highlights how collectivism fosters social harmony by prioritizing relationships grounded in morality and loyalty—to family, organizations, or society at large—over individual economic advantage (Cohen et al., 2016; Sun, 2008).

## Interaction between emotion and cultural values in pandemic

During the COVID-19 pandemic, these distinct cultural value orientations profoundly shaped both the daily lives and the psychological responses of populations across the globe.

Collectivism serves as a vital social buffer during periods of crisis; specifically, while pandemic-induced disruptions to daily life universally heighten feelings of social exclusion, this detrimental effect is significantly attenuated in collectivistic cultures compared to their more individualistic counterparts (Zhou et al., 2025). This social resilience underpins a broader protective effect on individual psychological states. Research consistently suggests that collectivist values bolster wellbeing and mitigate anxiety during upheaval (Ahuja et al., 2021; Ryff, 2014). This is further evidenced by Gong et al. (2022), who found that individuals endorsing collectivist norms reported higher wellbeing and lower anxiety regardless of their pandemic-related media consumption patterns. In contrast, those with weaker collectivist orientations experienced heightened distress even with limited media exposure, highlighting the fundamental role of cultural values in maintaining emotional stability.

Interestingly, while collectivism is linked to a heightened perception of infection risk—driven by stronger interpersonal connections and group consciousness—this perceived vulnerability does not necessarily translate into actual psychological maladjustment (Germani et al., 2020). Despite feeling more “at risk” due to their concern for the collective, individuals with strong collectivist ties often demonstrate greater emotional resilience.

However, this putative protective capacity appeared increasingly fragile in the later stages of the pandemic. Research on emotional expression during Shanghai’s 2022 citywide lockdown shows that public discourse was dominated by intense negative affect, including anxiety, anger, fear, and helplessness (Zhang et al., 2022; Zhao et al., 2024). These findings suggest that even in contexts often assumed to possess strong collectivist resources and social-resilience mechanisms, highly restrictive public health controls can impose substantial emotional strain and psychological burden on individuals.

This shift is plausibly rooted in the prolonged nature of containment policies and the chronic, cumulative stress they can generate. A broad body of work indicates that lockdowns are associated with overall deterioration in mental health and affective wellbeing, yet the pattern is rarely a simple linear drift toward “more negative” emotion. More commonly, stress-related indicators remain elevated over extended periods and exhibit renewed fluctuations as conditions change. In a multi-wave study spanning 1 year, Gori and Topino (2021) document precisely this combination of temporal variation and persistence: some symptoms change across phases, others remain relatively stable, and normative-psychological variables also shift, underscoring the staged and accumulative character of lockdown consequences.

Crucially, an emerging literature distinguishes between initial and subsequent lockdowns, emphasizing that prolonged or repeated restrictions can heighten fatigue and depletion. Using a repeated cross-sectional design comparing acute and prolonged phases, Shekriladze et al. (2022) show that as the stressor extends, mental health indicators worsen, plan-oriented coping declines, and the structure of coping shifts; associations that hold in the acute phase may attenuate or disappear under prolonged strain. This evidence supports a key inference for late-stage lockdown contexts: negative emotion in public discourse is not only more prevalent but also more likely to be expressed with greater intensity and sharper outward articulation. Converging longitudinal evidence from England’s household panel data likewise finds significant mental health impacts of repeated enforced lockdowns and points to the need for sustained psychological support (Dhensa-Kahlon et al., 2025). Together, these findings help explain why an unprecedented, large-scale lockdown in the third year of the pandemic could coincide with heightened negative affect in public discussion, and they motivate our decision to move beyond documenting the prevalence of negativity to examining the intensity with which negative emotion is expressed.

In China, cultural value orientations remain a significant source of variation in emotional intensity. While China is traditionally characterized as a quintessentially collectivist society, rapid economic growth has challenged the binary stereotype that equates East Asian cultures with collectivism and Western cultures with individualism. Recent research suggests that individualism levels in contemporary East Asian societies may have undergone a substantial rise (Akaliyski et al., 2025). In fact, individualism and collectivism are not mutually exclusive; rather, they often coexist within the same society or individual, with their relative salience determined by specific situational demands (Mo and Park, 2023). For instance, during the late-stage pandemic, heightened uncertainty and the erosion of social connectedness—driven by the closure of public spaces—may have further amplified the prominence of individualistic expression even within collectivist frameworks (Wang et al., 2024). The case of Georgia further illustrates this dynamic of cultural hybridization: although the country is conventionally viewed as highly collectivist, social transformation and globalization have fostered a mixed cultural landscape that became particularly visible during the pandemic (Shekriladze et al., 2021). This suggests that even within ostensibly collectivist contexts, emotional responses exhibit significant differentiation driven by the dynamic evolution of cultural values.

In the Chinese context, Xiao (2021) further refines the individualism–collectivism framework by distinguishing its horizontal and vertical dimensions. In this typology, horizontal individualism emphasizes autonomy under conditions of equality, whereas vertical individualism combines autonomy with competition and status differentiation. Horizontal collectivism stresses interdependence and cohesion among relatively equal group members, whereas vertical collectivism combines interdependence with acceptance of hierarchy, authority, and unequal roles within the group (Triandis et al., 1988). This four-factor framework shows that value orientations, which range from peer-based autonomy and cohesion to status-based competition and obligation, systematically shape individuals’ attitudes toward protective recommendations, levels of worry, and broader psychological responses during public health crises.

For the present study, this perspective is particularly consequential: when the outcome of interest is negative emotion intensity, cultural orientation should be treated as a mechanism that reshapes stress appraisal and relational experience, not merely as a categorical marker used to compare “more positive” versus “more negative” sentiment. This framing also reinforces our central empirical claim that late-stage lockdown discourse is especially prone to intensified, potentially spilling-over emotional expression—making intensity, rather than valence alone, the more diagnostic outcome in a 2022 lockdown setting. Building upon the premise that value orientations within a society can diverge and manifest differently depending on the context, this study first aims to clarify the general relationship between cultural values and the intensity of negative emotions.

However, given that public health crises involve a diverse array of concerns (Zhao et al., 2020), the specific discussion topic may serve as a critical boundary condition for this relationship. Previous research has identified distinct themes in COVID-19 discourse (Lu Y. et al., 2021) and observed that emotional responses vary significantly across different subjects (Chen and Xu, 2024). Consequently, this research investigates whether the moderating effect of cultural values is sensitive to the specific topic under discussion.

The study proposes the following research questions:

*RQ1*: How do cultural value orientations (individualism and collectivism) influence the intensity of negative emotions expressed on Chinese social media during the during the 2022 Shanghai lockdown?

*RQ2*: How do different discussion topics moderate the relationship between these cultural value orientations and the intensity of negative emotions?

## Method

### Case selection

The 2022 Shanghai lockdown serves as a representative case for this study due to its unprecedented scale and policy stringency. The city implemented city-wide “static management” from March 28 to June 1, 2022, a period marked by over 600,000 infections (Cao, 2022; Zhou and Zhu, 2022). This wave’s epidemic scale exceeded that of the initial 2020 Wuhan outbreak (Liu and Du, 2022). During this window, the city enforced the “three pauses” (suspension of in-person work, non-essential business operations, and taxi/ride-hailing services) and the “three no’s” (no gathering, no movement, and no leaving home) (The Paper, 2022).

The intensity of the restrictions created a high-pressure environment that polarized public discourse regarding the epidemic prevention measures and the steadfast strategy of refusing to “live with the virus” (Lamb, 2022). Furthermore, the prolonged isolation necessitated by these policies forced a confrontation between collective public health responsibilities and individual freedoms. This offers a naturalistic context to investigate emotional responses to public health policies and their underlying cultural dimensions.

### Data collection

Data for this study were collected from Zhihu, a major Chinese question-and-answer platform similar to Quora, where users share and seek knowledge, often perceived as professional and trustworthy. On Zhihu, users can initiate discussion threads by posing questions or raising topics, and other users can participate by providing answers or posting comments. Users have the option to post anonymously.

Research indicates that Zhihu’s user base is predominantly middle-class, with approximately 80% holding a bachelor’s degree or higher (Zhang, 2020). Through discussions on socio-political topics, Zhihu has become a significant platform for digital civic engagement in China (Peng et al., 2022). Since the onset of the COVID-19 pandemic, numerous discussions concerning China’s containment policies have taken place on Zhihu. Moreover, compared to other Chinese social media platforms such as Weibo (a microblogging service similar to Twitter), posts on Zhihu tend to be longer, and users often engage in more detailed explanation and analysis rather than brief assertions (Ru and Hu, 2016). Consequently, Zhihu discussions represent valuable data for the present research.

To gather a comprehensive dataset related to the dynamic zero-COVID policy, we searched Zhihu using the keywords “dynamic zero-COVID” and “virus, coexistence” and employed Python scripts to crawl all questions and answers returned by these searches. The data crawling process was completed on June 8, 2022. This process yielded 70 relevant discussion threads containing a total of 7,687 answers.

To ensure the analysis precisely reflected the discourse during the lockdown phase, we filtered the data according to the official beginning and ending dates of the Shanghai lockdown. Specifically, we retained only those posts published between March 28, 2022 and June 1, 2022. Following this rigorous temporal filtering, along with deduplication and the removal of blank entries, a final dataset of 3,881 unique texts remained for analysis.

Following data compilation, the text corpus was cleaned by removing HTML tags, emojis, and non-informative content such as advertisements or boilerplate text. Punctuation, personal pronouns, and function words were preserved, as they can provide important semantic cues for the subsequent analytical steps.

### Cultural value orientations labeling

To identify cultural value orientations within the discourse, we focused on the contrasting constructs of collectivism and individualism (refer to Table 1). Drawing upon Hofstede’s cultural dimensions framework and pertinent literature on cross-cultural values, we developed a thematic lexicon designed to capture linguistic and conceptual indicators specific to each orientation (details in Table 2). This lexicon comprises five analytical categories, each representing a distinct class of discourse features useful for differentiating between collectivist and individualist expressions:

**Table 1:** Dimension of individualism and collectivism.

Dimension	Individualism	Collectivism
Core emphasis	Personal goals, freedom, rights, and independence	Group or societal interests, harmony, responsibility, and obligation
Typical language cues	“I,” “my choice,” “personal decision,” “individual freedom”	“We,” “group interests,” “teamwork,” “social responsibility,” “collective goal”
Underlying value judgement	Focus on self-realization and maximizing individual benefit	Focus on common good, social harmony, and overall wellbeing

**Table 2:** Typical terms and themes of collectivism and individualism.

Category	Collectivism	Individualism
Pronouns	We, everyone, group, collective, society, team	I, individual, self, my decision, individual freedom
Responsibilities and obligations	Shared responsibility, social responsibility, mutual aid, solidarity, sacrifice of personal interests	Personal choice, right to privacy, freedom of speech, individual needs
Goals and core concepts	Overall welfare, public interest, common goal, group value	Self-realization, maximization of personal interest, personal goal
Discourse features	Emphasizes unity, mutual support, safeguarding common interests	Emphasizes personal will, independence, individual responsibility (not collective constraints)
Moral inclination	Focuses on contributing to others or society, positive value of altruism	Focuses on self-development, personal success, individual enjoyment or rights

The process for labeling posts according to value orientation combined automated analysis with manual adjudication to ensure both scalability and reliability. A Python script was used to tokenize each text and calculate two raw scores based on the frequency of keywords from the lexicon: a Collectivism Score and an Individualism Score. Initially, each of the five analytical dimensions was equally weighted in the score calculation. Posts exhibiting a score difference of two or more between the two orientations were automatically labeled as either “Collectivist” or “Individualist.”

A Python script tokenises each post and counts keyword hits. For each post, two raw scores were calculated:


$$\text{Collectivism}\_\text{score}=\sum_{d=1}^{5}w_{d}\times {\text{hits}}_{d,\text{collevtivism}}$$ (1)

$$\text{Individualism}\_\text{score}=\sum_{d=1}^{5}w_{d}\times {\text{hits}}_{d,\text{individualism}}$$ (2)

In Equations 1, 2, w_d_ represents the predefined weight assigned to each dimension as specified in Table 2 (currently set to 1), and hits_d, Collectivism_ denotes the count of keywords related to the respective cultural value orientation (collectivism or individualism) identified within dimension. Posts exhibiting a significant difference between the two scores (e.g., a difference of ≥ 2 hits) were automatically labeled, while posts with ambiguous scores (e.g., tied scores or low scores for both orientations) were flagged for manual review.

Additionally, text entries characterized by low or balanced scores—such as tied frequencies, low overall keyword density, or the co-occurrence of keywords from opposing value orientations—were also flagged for manual review. Two bilingual coders independently assessed each flagged post, considering contextual factors including sarcasm, negation, narrative framing, and rhetorical devices. Disagreements between coders were resolved through discussion, and inter-coder reliability was assessed using Cohen’s Kappa. The resulting Cohen’s Kappa coefficient of 0.82 indicated substantial agreement, ensuring consistent labeling.

Following this process, each post was assigned a final label: Collectivist (predominantly reflecting collective values) or Individualist (predominantly reflecting personal values). The original numerical scores were retained to facilitate further quantitative analyses, such as regression modeling with sentiment metrics or clustering based on value orientation.

### Sentiment analysis

Recent research has highlighted the strong performance of large language models (LLMs) across various text annotation tasks, including sentiment analysis (Törnberg, 2023; Wang et al., 2023; Ziems et al., 2024; Zhang et al., 2023). This study utilized the “gpt-3.5-turbo” model to classify the intensity of negative emotion expressed in 3,881 social media posts. Emotion intensity was categorized on a three-point scale: 1 = Not Negative, 2 = Negative, and 3 = Strongly Negative. To ensure deterministic output and consistency, the model’s temperature parameter was set to 0.

Although LLMs demonstrate reliable performance in basic sentiment polarity classification (e.g., positive/negative/neutral) (Wang et al., 2023; Wei and Liu, 2025), their effectiveness can be limited in more nuanced tasks like classifying ordered levels of emotional intensity (Zhang et al., 2023). To enhance performance for intensity classification, this study employed a few-shot learning strategy, embedding example texts pre-labeled for each intensity level directly within the prompt to guide the model towards more accurate and consistent labeling.

The prompt designed for this task comprised three core components: (1) a contextual description of the text data source, (2) explicit classification instructions, and (3) illustrative example texts, previously labeled by human coders, for each level of emotional intensity. The complete prompt structure was as follows:

*“These texts were posted between March and June 2022, during Shanghai’s static management period prompted by the COVID-19 outbreak. They are drawn from social media discussions concerning China’s dynamic zero-COVID policy. The data are intended for analyzing users’ emotional expressions.*

*Assessing the intensity of negative emotion in these texts will help us better understand and evaluate the public’s emotional responses to pandemic control policies and the lived experience during this period.*

*Label definitions:*

*1: Not negative.*

*2: Negative.*

*3: Strongly negative.*

*Text: Whatever policies the central government issues, I fully support and follow them. If the state tells me to quarantine, I quarantine. If the state says zero-COVID is good, then zero-COVID is good. If the state says coexistence is good, then coexistence is good. I follow whatever the state says.*

*Label: 1.*

*…*

*Please classify the following text according to the intensity of negative emotion, and provide only the label as output:*

*…”*

To validate the LLM’s labeling performance, two authors independently annotated a randomly selected test subset of 200 posts. Inter-coder reliability between these human annotators achieved a Cohen’s Kappa of 0.84, signifying substantial agreement. Using this few-shot learning setup (providing four examples per intensity level, see Table 3), the GPT model achieved an accuracy of 75.5% and a macro-average F1 score of 0.75 when evaluated against the human-labeled test set. These performance metrics were deemed acceptable for the subsequent analyses in this research.

**Table 3:** Examples of social media texts by level of negative emotion intensity.

Example text	Negative emotion intensity
Whatever policies the central government issues, I fully support and follow them. If the state tells me to quarantine, I quarantine. If the state says zero-COVID is good, then zero-COVID is good. If the state says coexistence is good, then coexistence is good. I follow whatever the state says.	1 (not negative)
They say no one is dying or getting sick abroad, but in fact, hospital beds are running out, people have to buy their own medicine, and if complications aren’t handled well, there can even be mental health consequences.	2 (negative)
I heard that COVID can cause impotence and brain atrophy!!! If you are so eager to coexist with the virus, just buy a plane ticket and go live with it abroad!	3 (strongly negative)

### Text clustering

The study utilized the *K*-means unsupervised clustering algorithm to group the 3,882 texts based on content similarity. Texts were represented using word embeddings (vectors) to capture semantic features, enabling the algorithm to assign similar texts to the same cluster. The optimal number of clusters was determined using the elbow method, which analyzes the sum of squared errors (SSE) across varying numbers of clusters (k) (Su and Dy, 2004). The dominant topic for each resulting cluster was identified by examining its representative keywords and sample texts.

Following these text analysis procedures, the study derived key variables for subsequent modeling: two categorical predictors (cultural value orientation and discussion topic) and one ordinal outcome variable (negative emotion intensity). These variables formed the basis for the subsequent regression analyses.

## Results

### Descriptive statistics

#### Negative emotion intensity and cultural value orientations

Among the 3,881 analyzed posts, 1,923 were classified as reflecting collectivist cultural values, and 1,958 were classified as reflecting individualist orientations. Regarding emotion intensity, the LLM annotations indicated that public discourse concerning the dynamic zero-COVID policy during Shanghai’s lockdown period (referred to as “static management”) was predominantly negative. Overall, 80.2% of the posts were labeled as either “Negative” or “Strongly Negative” (Table 4).

**Table 4:** Frequency and percentage distribution of key variables.

Variable type	Variable	Category	Total (*N*)	Individualism *n* (%)	Collectivism *n* (%)
Predictor variables	Cultural value orientations	—	3,881	1958 (50.5%)	1923 (49.5%)
	Topic	Medical resources	833	357 (42.9%)	476 (57.1%)
		Implementation of anti-COVID19 measures	2,547	1,273 (50.0%)	1,274 (50.0%)
		General scientific knowledge	131	93 (71.0%)	38 (29.0%)
		Daily work and life	370	235 (63.5%)	135 (36.5%)
Outcome variables	Negative emotion intensity	Not negative	767	468 (61.0%)	299 (39.0%)
		Negative	1784	909 (51.0%)	875 (49.0%)
		Strongly negative	1,330	581 (43.7%)	749 (56.3%)

#### Negative emotion intensity and topics

The texts were clustered into four categories (Table 5). Cluster 1 (Largest): Comprised texts discussing the perceived benefits of COVID-19 control measures alongside criticisms or issues regarding their implementation by administrative personnel. Cluster 2: Contained texts primarily expressing concerns about potential shortages and the uneven distribution of medical resources under the “dynamic zero-COVID” policy. Cluster 3: Included discussions on the feasibility of virus eradication, drawing from scientific, biological, and historical perspectives. Cluster 4: Focused on personal narratives detailing individuals’ living and working conditions during the pandemic, with users sharing everyday experiences from various locations. Table 5 displays keywords with high weights and representative sample texts for each identified discussion topic.

**Table 5:** Results of texts’ *K*-means clustering.

Category	Feature words	Text sample
Medical resources	Easing, deaths, medical, vaccines, mortality, resources, hospitals, intensive, data, numbers etc.	小城市及农村医疗资源非常匮乏, 国家的医疗投入总体不足。如果农村被大面积感染, 的确是非常头疼的事情…… (Medical resources, in small towns and rural areas are very scarce, and the state’s investment in health care is generally inadequate. It would indeed be a great problem if people in rural areas become largely infected.)
Implementation of anti-COVID-19 measures	Support, life, policy, nucleic acid, quarantine, anti-pandemic, cold, abyss of misery, flu, elderly etc.	基层工作人员鸡毛当令箭, 省市区街道层层加码, 这些问题需要有统一明确的规定…… (With authorization, grassroot officials use their power to the utmost by emposing more epidemic control measures level by level from province right down to the community. Problems like this need to be solved.)
General scientific knowledge	Human, eradication, smallpox, history, science, logic, evolution, time, mutation, mortal combat etc.	人类生存繁衍的历史, 从一定意义上讲, 也是一部与病毒拼死相争的历史, 不是人类消灭病毒, 就是人类被病毒吞噬…… (The history of human evolution and reproduction is, in a certain sense, also a history of desperate struggle with viruses, either human beings destroy viruses or human beings are devoured by viruses.)
Daily work and life	Masks, coordinates, work, friends, home, out, company, school, work, mandatory etc.	坐标新加坡。现在出门已经可以不戴口罩了。室内大家还是都带着口罩。餐馆也爆满了…… (Located in Singapore. It is now possible to go out without a mask. But everyone still wears a mask indoors. Restaurants are also full.)

**Figure 1:**
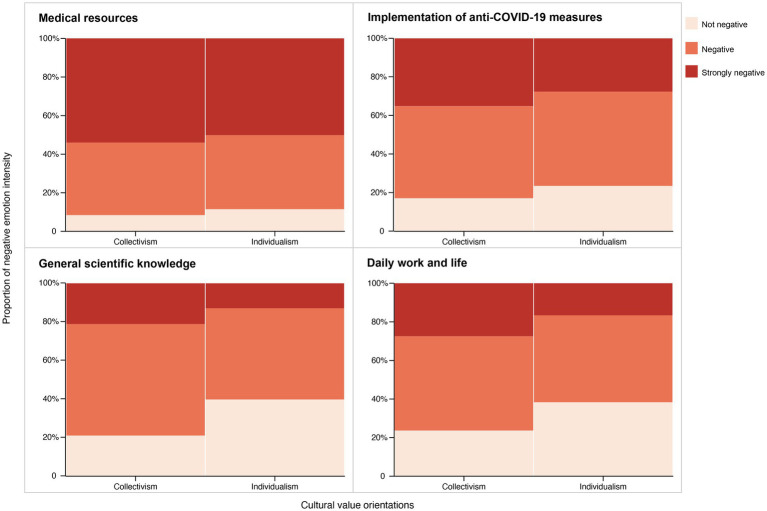
Distribution of negative emotion intensity across different cultural value orientations and topics.

As illustrated in Figure 1, the distribution of negative emotion intensity differed significantly across the identified topics. Sentiment related to “*medical resources*” was the most intensely negative, exhibiting a significantly higher proportion of posts classified as “Strongly Negative.” Conversely, discussions concerning *scientific knowledge* and *daily work and life* experiences displayed more moderate levels of negative emotion. A noteworthy pattern observed across all topics was that posts reflecting collectivist cultural values consistently displayed a higher intensity of negative emotion, particularly within the “Strongly Negative” category.

### Ordinal logistic regression

As the outcome variable is ordinal, this study utilized an ordinal logistic regression model to examine the effects of cultural value orientation and discussion topic on this variable. In the model specification, “Collectivism” was designated as the reference category for cultural value orientation, and “medical resources” served as the reference category for discussion topic.

The proportional odds assumption, a prerequisite for this model, was assessed using the oparallel command in Stata. Results from the Wolfe-Gould test (*χ*^2^(7) = 2.51, *p* > 0.05), the Brant test (*χ*^2^(7) = 2.38, *p* > 0.05), and the likelihood ratio test (*χ*^2^(7) = 2.49, *p* > 0.05) were all non-significant. These findings confirm that the proportional odds assumption was met, indicating that the effects of the predictors are consistent across the different cut-points of the ordinal outcome variable.

Table 6 summarizes the results of the ordinal logistic regression model. For each predictor, the table presents the estimated log-odds coefficients (*β*), corresponding odds ratios (OR), and significance levels. The main effect coefficient for cultural value orientation was not statistically significant (*β* = −0.19, *p* = 0.17, OR = 0.83). This suggests that within the reference topic (*medical resources*), cultural value orientation did not significantly predict the intensity of negative emotion.

**Table 6:** Ordinal logistic regression model estimating the effect of cultural value orientations and topic on the negative emotion intensity.

Predictors	*β* (SE)	Odd ratios (LCI, UCI)
Cultural value orientations (Ref: Collectivism)		
Individualism	−0.19 (0.14)	0.83 (0.64, 1.08)
Topic (Ref: Medical resources)		
Implementation of anti-COVID-19 measures	−0.78 (0.10)***	0.46 (0.38, 0.56)
General scientific knowledge	−1.25 (0.31)***	0.29 (0.16, 0.53)
Daily work and life	−1.15 (0.19)***	0.32 (0.22, 0.45)
Topic# cultural value orientations		
Implementation of anti-COVID-19 measures * individualism	−0.18 (0.15)	0.83 (0.62, 1.13)
General scientific knowledge * individualism	−0.52 (0.38)	0.59 (0.28, 1.25)
Daily work and life * individualism	−0.50 (0.25)*	0.61 (0.38, 0.99)

*p < 0.05, **p < 0.01, ***p < 0.001.

**Figure 2:**
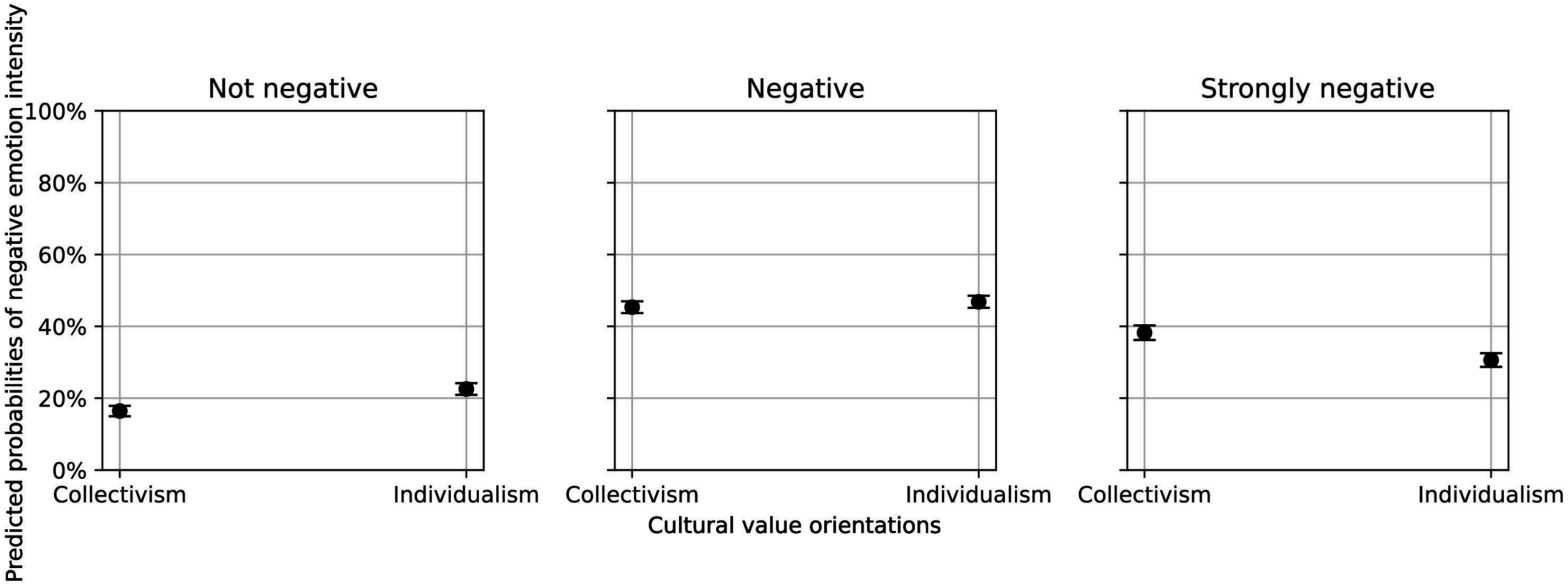
Predicted probabilities of negative emotion intensity across cultural value orientations.

However, further analysis using average marginal effects revealed significant differences in predicted probabilities across the three levels of emotional intensity. Compared to collectivist texts, those reflecting individualist values showed a 6.13 percentage point higher predicted probability of expressing neutral or positive sentiment (*p* < 0.001), and a 1.5 percentage point higher predicted probability for moderately negative sentiment (*p* < 0.05). Conversely, collectivist texts exhibited a 7.6 percentage point higher predicted probability of expressing strongly negative emotion (*p* < 0.001). Figure 2 visualizes these differences in predicted probabilities based on cultural orientation.

Regarding topic effects, discussions concerning medical resources were most strongly associated with negative emotional expression among the variables analyzed. Specifically, the odds ratio (OR) for the topic “implementation of anti-COVID-19 measures” was 0.46 (*p* < 0.001), indicating that the odds of expressing more intense negative emotion were 54% lower for this topic compared to the baseline topic (*medical resources*). Similarly, the topics “*scientific knowledge*” and “daily life and work” had odds ratios of 0.29 (*p* < 0.001) and 0.32 (*p* < 0.001), respectively, both significantly reducing the odds of more negative emotional expression (see Table 6).

**Figure 3:**
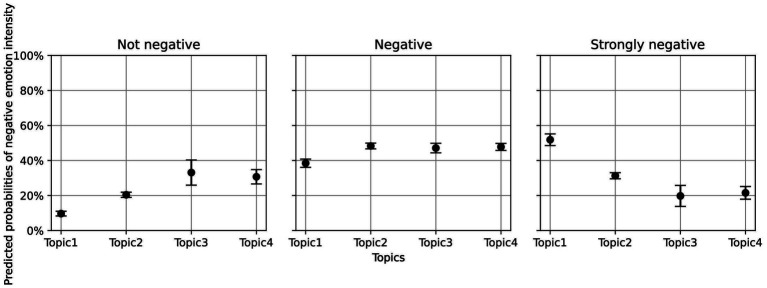
Predicted probabilities of negative emotion intensity across different topics. Topic 1 = “Medical resources”; Topic 2 = “Implementation of anti-COVID19 measures”; Topic3 = “General scientific knowledge”; Topic4 = “Daily work and life”.

Figure 3 illustrates the predicted probabilities associated with each topic. Compared to the baseline, the likelihood of strongly negative emotion was significantly lower for the topics “implementation of anti-COVID-19 measures” (*Δ* predicted probability = −0.21, *p* < 0.001), “*scientific knowledge*” (Δ = −0.32, *p* < 0.001), and “daily life and work” (Δ = −0.30, *p* < 0.001), reaffirming that discussions of “*medical resources*” were most prone to heightened negative emotion intensity.

*Post-hoc* pairwise comparisons further revealed that, in addition to differences from the baseline topic, a significant contrast also emerged between the topic “Implementation of anti-COVID-19 measures” and “daily life and work”(*β* = 0.38, *p* < 0.05, OR = 1.46), with the former being 1.46 times more likely to trigger higher levels of negative emotion than the latter.

The effect of cultural value orientations on the negative emotion intensity was also moderated by topics. This moderation was most evident in the topic “daily life and work,” which amplified the influence of cultural values (*β* = −0.50, *p* < 0.05, OR = 0.61; see Table 6). Specifically, for individualist texts, the odds of expressing more intense negative emotion was further reduced by 39% in this topic compared to the baseline topic of “*medical resources*.” Pairwise comparisons of this moderation effect for the other topics relative to the baseline were non-significant, suggesting that the influence of cultural values on emotional intensity did not differ significantly between those topics.

**Figure 4:**
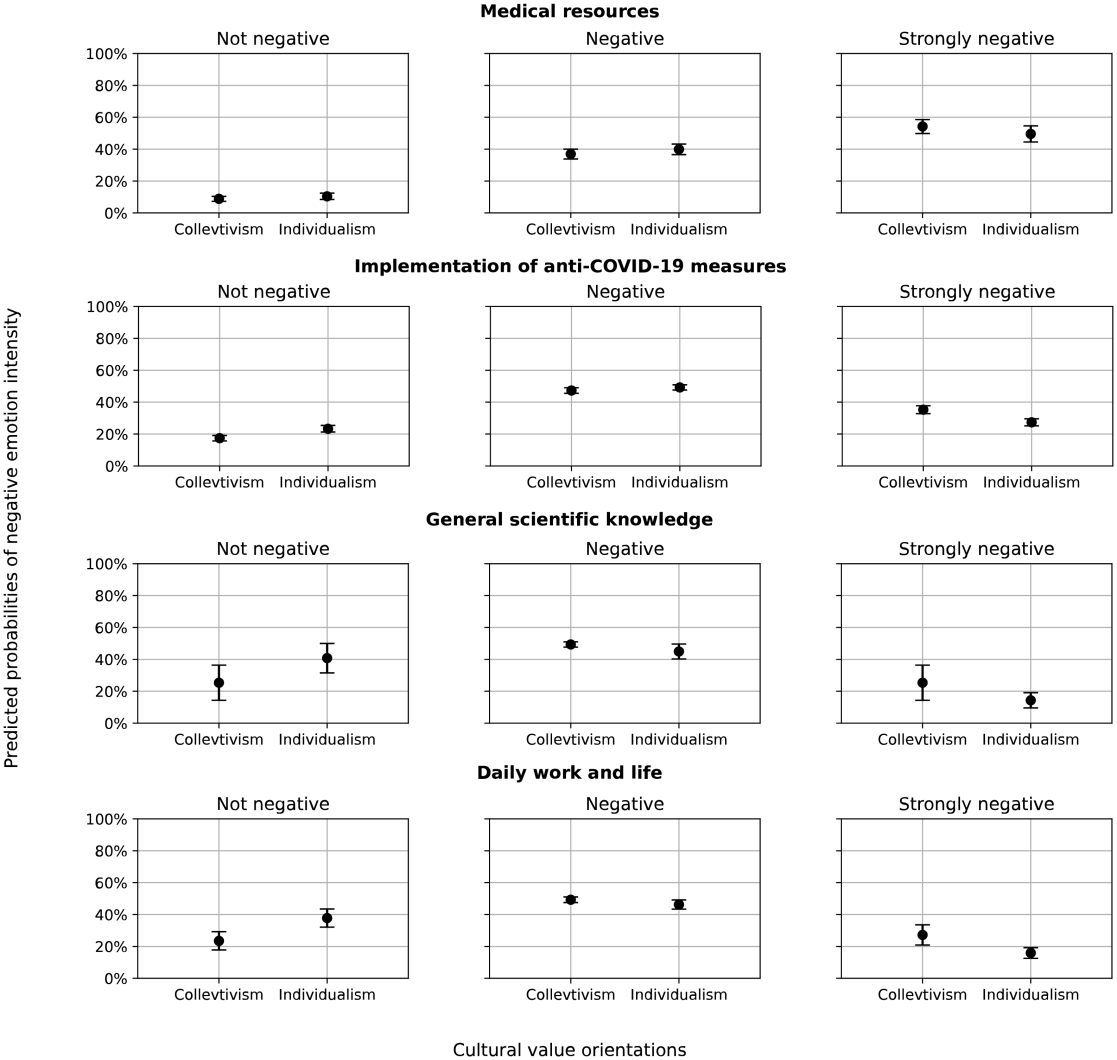
Predicted probabilities of negative emotion intensity across different topics and cultural value orientations.

However, further analysis of conditional marginal effects (Table 7) showed that, even though most topics did not exhibit significant differences in their influence on the effect of cultural value orientations, a consistent pattern emerged within the topic “Implementation of anti-COVID-19 measures” and “daily life and work”: individualist orientations were associated with a significantly higher predicted probability of non-negative emotion, while collectivist orientations were linked to a higher probability of strongly negative emotional expression. This effect was particularly pronounced in the topic “daily life and work,” where the differences in predicted probabilities for non-negative and strongly negative emotion reached 14.3% (*p* < 0.01) and 11.3% (*p* < 0.01), respectively. Figure 4 visualizes these predicted probabilities of emotional intensity, broken down by topic and cultural value orientation.

**Table 7:** Conditional marginal effects of cultural value orientations on negative emotion intensity.

Topic	Outcome level	Conditional effect	SD
Implementation of anti-COVID-19 measures	Not negative	0.060***	0.012
	Negative	0.019***	0.005
	Strongly negative	−0.079***	0.016
Daily work and life	Not negative	0.143**	0.041
	Negative	−0.030*	0.013
	Strongly negative	−0.113**	0.036

*p < 0.05, **p < 0.01, ***p < 0.001.

## Discussion

Set within the context of the three-month “static management” period during the 2022 COVID-19 outbreak in Shanghai, this study examines how cultural values influence the intensity of negative emotional expression on social media, and how this relationship is moderated by discussion topics. Data were drawn from Zhihu, a prominent Q&A-based social media platform in China.

Regarding emotional expression, the findings indicate a predominantly negative tone in social media discourse during the Shanghai lockdown, with a significant proportion of texts conveying strong negative emotion. Notably, while waves of negative sentiment (e.g., fear, sadness, anxiety) were observed on Chinese social media during earlier phases of the pandemic (Li S. et al., 2020; Zhao et al., 2020; Zheng et al., 2024), and subsequent periods saw returns to more neutral or positive states (Li Q. et al., 2020; Zhao et al., 2020; Zheng et al., 2024), our findings indicate that the extended Shanghai lockdown in 2022 led to a renewed deterioration in public emotional wellbeing.

This shift in emotional tone may relate to the evolving focus of public discourse throughout the pandemic. During early stages, the public actively sought information to comprehend the collective health threat; unmet informational needs often fueled negative emotions like anxiety and fear. As the situation progressed, social media increasingly featured news updates, factual reports, and guidance on protective measures, which seemingly helped alleviate public concerns and foster a more neutral or positive emotional climate (Zhao et al., 2020; Zhao and Basnyat, 2022). However, by 2022—the third year of the pandemic—public attention had shifted. Our study finds discussions increasingly centered on the implementation of control policies, medical resource allocation, and disruptions to daily life and work. This aligns with previous research (Chen and Xu, 2024), suggesting that during prolonged public health crises, anxieties about personal health risks may yield to broader concerns about the sustainability of daily life and social functioning.

Second, regarding the influence of cultural value orientations, this study confirms significant differences between texts reflecting individualism and collectivism in their expression of negative emotion intensity. While individualist texts were marginally more likely to express any negative emotion, collectivist texts were notably more prone to conveying strongly negative emotional content. This finding diverges somewhat from prior research suggesting collectivism acted as a buffer against psychological distress during the pandemic’s early stages, particularly under nationwide lockdowns, potentially making individuals less susceptible to stress, difficulty, and anxiety (Germani et al., 2020).

Germani et al. (2020) attributed this buffering effect to the tendency for individualists to perceive the self as stable and the environment as changeable; when faced with uncontrollable circumstances like strict public health measures, they might struggle to adapt, increasing psychological strain. Conversely, collectivists may view adaptation as a moral duty, willingly modifying behavior for collective needs (Triandis, 2001). However, our study focuses on the third year of the pandemic, a period marked by growing dissatisfaction with prolonged, strict controls. Under these circumstances, collectivists might have experienced heightened distress not solely due to the policies themselves, but potentially in response to perceived violations of social duty by others—particularly individualists seen as neglecting obligations or resisting compliance. Examples of such sentiments from the data include:

I absolutely do not support coexisting with the virus—coexistence is nothing more than a cowardly excuse. If the rest of the world had imposed strict controls like China from the beginning, would the virus still be this rampant today? Now a bunch of incompetent people are calling for coexistence—what a ridiculous notion.

A friend of mine said they support coexistence, and I replied: if your elderly or children cannot make it through, will you take responsibility? If my elderly or children cannot make it through, will you be accountable? …

Third, the influence of discussion topic on emotional expression was also significant. The study revealed that discussions centered on *the* “implementation of anti-COVID-19 policies” were associated with particularly intense negative emotion. This aligns with previous research finding that public discourse on pandemic prevention policies often involves intense negative emotions, including skepticism, resistance, and distrust towards the measures themselves (Chen and Xu, 2024).

However, the topic most strongly associated with negative emotional responses was “*medical resources*.” For collectivist-oriented texts, concerns often centered on the fear that a large-scale outbreak would overwhelm the healthcare system:

Our current hospital bed capacity is barely sufficient for regular medical needs, and now we have to free up beds for a large number of critical COVID cases…

In contrast, individualist-oriented texts were more likely to express frustration over the perceived misallocation of resources towards pandemic control at the expense of other patients:

When will we finally protect hospitals—so that limited medical resources can actually go toward helping critical patients? Healthcare resources have been consumed by COVID measures. Everyone is suffering. People who truly need care are being locked out of hospitals.

Finally, this study highlights the moderating role of discussion topics on the relationship between cultural values and negative emotion intensity, which constitutes a primary contribution of our work. The topic of “daily life and work” functioned as a significant moderator by substantially amplifying the influence of cultural orientations. Specifically, within this thematic context, collectivist-oriented discourse was significantly more likely than individualist-oriented content to express high-intensity negative affect.

Beyond the daily life topic, the category of “Implementation of anti-COVID-19 measures” also exhibited distinct conditional marginal effects. However, the emotional pattern here was unique. Rather than a generalized increase across all negative levels, collectivist discourse was specifically characterized by an increased probability of expressing strong negative emotions.

This heightened negativity among collectivists across these two topics likely stems from their fundamental prioritization of collective interests and their corresponding adherence to public health mandates (Chen et al., 2021; Mo and Park, 2023; Wei et al., 2024). As reflected in our dataset, collectivist expressions often embodied a staunch defense of China’s dynamic zero-COVID policy and its rigorous containment strategies. Consequently, when segments of the public began to exhibit fatigue or skepticism in the later stages of the pandemic, a phenomenon that effectively manifested as “moral disengagement” through deviations from shared behavioral norms, collectivists responded with intensified negative affect. This reaction likely represents a form of moral indignation toward perceived threats to collective safety.

The topic of “daily life and work” further illustrates this cultural divergence through the lens of global pandemic experiences. While this category encompasses users from various regions sharing their personal routines, these narratives are fundamentally situated within the vastly different anti-epidemic policies of their respective countries. Consequently, even discussions of daily activities inevitably center on the impact of these policies. Our analysis shows that collectivist-oriented texts frequently voiced discomfort with the relaxation of restrictions in other regions while advocating for continued adherence to domestic policies to protect vulnerable groups:

Germany fully reopened in April. I still do not want to get infected, but I guess it’s inevitable at some point. With three elderly family members back home still unvaccinated, I really cannot say I support reopening.

Conversely, individualist-oriented texts in this context displayed more moderate or even positive emotional tones. They appeared more inclined to prioritize the restoration of personal agency and the freedom of movement over the collective infection risk. These individuals viewed the return to “normal life” as a primary buffer against pandemic-related stress:

Based in the UK. Life has pretty much returned to normal, apart from the fact that I still cannot go back to China.

This contrast underscores how cultural values shape the diverse ways in which individuals interpret the relationship between personal autonomy and collective commitments, which ultimately dictates the intensity of their emotional expression during a public health crisis.

This study makes theoretical contributions by moving beyond the predominantly descriptive analyses common in early COVID-19 social media research. First, situated in the unique context of Shanghai’s strict 2022 lockdown—a later stage of the pandemic featuring engagement and clashes between individuals with divergent cultural orientations—it reveals that even with widespread public familiarity with the virus and protective measures, prolonged restrictions can generate substantial negative emotion due to shifting public concerns. Second, by introducing cultural value orientation as a key predictor, the study advances an explanatory understanding of factors driving negative emotion intensity. In identifying the moderating role of discussion topics, it further enriches theoretical perspectives on how varying degrees of negative emotional expression emerge during protracted public health crises, offering a new analytical lens for future research on digital emotions and value-driven communication in emergencies.

From a practical standpoint, this study offers an empirical foundation for refining risk communication strategies during public health crises. Its findings can enable government agencies to better anticipate the concerns and expectations of diverse public segments across various stages of a crisis. By identifying high-risk topics and value-driven audience groups prone to heightened emotional distress, policymakers and media organizations can develop more targeted, timely, and transparent messaging. Such tailored communication can help mitigate extreme emotional responses and foster more effective public opinion management.

## Conclusion

In summary, this study analyzed 3,881 Zhihu posts from Shanghai’s 2022 ‘static management’ period using GPT-3.5-based emotion intensity annotation, a lexicon-based classification of cultural value orientations, and *K*-means topic clustering. Findings reveal a high level of negativity in public discourse overall. Cultural value orientations significantly influenced negative emotion intensity: texts reflecting collectivist values were more prone to high-intensity negative emotion, whereas those reflecting individualist values tended toward neutral or positive expression. Furthermore, topics concerning “*medical resources*” and “Implementation of anti-COVID-19 measures” were associated with more intense negative emotion. Topic also moderated the effect of cultural values, with the topic “daily life and work” amplifying emotional differences between value orientations. These results integrate the individualism–collectivism framework into emotion research, address a gap concerning emotional intensity, and offer practical insights for differentiated communication strategies during crises.

Taken together, prior evidence and our findings support a revised account of how intense negative emotion emerges under prolonged restrictions. Sustained lockdown conditions can convert uncertainty and resource strain into a chronic background rather than an episodic shock. As a result, public emotions are not merely immediate reactions to discrete events; they also reflect cumulative experiences of extended constraint and repeated policy recalibration. Research on repeated lockdowns indicates that as the stressor persists, psychological functioning is more likely to shift toward fatigue and depletion, accompanied by a structural reconfiguration of coping repertoires: coping strategies that are more planful and action-oriented in acute phases may weaken under prolonged strain (Dhensa-Kahlon et al., 2025; Shekriladze et al., 2022). In this extended phase, emotion-focused coping—particularly seeking and providing affective support—may become more consequential, insofar as it helps buffer chronic stress and enables individuals to manage exhaustion and diminished control.

Against this backdrop, the value-orientation differences observed in our data can be understood as distinct modes of meaning-making within the same high-pressure environment. Collectivist-oriented discourse foregrounds shared obligation and normative order; when discussions center on high-stakes issues such as medical resources or policy implementation, this orientation more readily invites moralized appraisal and forceful expression, thereby elevating negative emotion intensity. Individualist-oriented discourse, by contrast, places greater emphasis on personal boundaries and self-regulation, and is therefore more likely to manifest as neutralized or comparatively restrained affective expression. This interpretation aligns with prior evidence that cultural orientations enter the coping–emotion nexus as process variables rather than mere background traits (Shekriladze et al., 2021; Xiao, 2021). It also resonates with cross-cultural findings showing that cultural context conditions how perceived disruption translates into relational experiences, such as social exclusion, which in turn shapes emotional expression (Zhou et al., 2025).

### Limitation and future study

It is crucial to acknowledge several limitations that contextualize the findings of this study. First, regarding the data source and collection, the analysis relies exclusively on data from Zhihu. While Zhihu serves as a premier intellectual platform in China, the social media ecosystem is highly fragmented and diverse. The absence of cross-platform comparisons limits the generalizability of these results to other environments, such as Weibo, where user demographics, censorship intensities, and discourse norms may vary significantly.

Second, a methodological constraint involves the modality of the data analyzed. The current annotation of emotion intensity was confined to textual content, thereby overlooking non-textual elements such as images, emojis, and memes, which are increasingly prevalent and emotionally potent in contemporary online discourse. Future research would benefit from incorporating multimodal analysis to more comprehensively capture the full emotional landscape of digital communication.

Third, extending from the data modality to the conceptual measurement, the emotional categorization adopted in this study focused primarily on the intensity of negative affect. By classifying emotions as non-negative, negative, and strongly negative, this unidirectional approach represents a conceptual limitation, as it collapses neutral and positive sentiments into a single “non-negative” category. This bias toward negative intensity may have precluded the identification of how cultural values influence the expression of optimism. Future research should employ a more balanced, bidirectional emotional scale to explore whether cultural orientations exert a similar moderating effect on the expression of positive emotions. In addition, because our value-orientation measure is inferred from language in posts rather than respondents’ household contexts, we cannot directly test whether caregiving responsibilities or concern for extended-family welfare account for the stronger negative intensity observed in collectivist-oriented discourse. Future work could operationalize caregiving-related cues (e.g., references to elderly care, dependent family members, and caregiving tasks), incorporate household-structure indicators where ethically feasible, or link platform text to survey-based measures to adjudicate this mechanism more directly.

Finally, a significant limitation arises from the uneven distribution of statements across topic categories. While the “*Implementation of anti-COVID-19 measures*” category provided a robust volume of data, the categories of “*Daily work and life*” (*n* = 370) and “g*eneral scientific knowledge*” (*n* = 131) had comparatively smaller sample sizes. In the case of “g*eneral scientific knowledge*,” the limited sample likely resulted in insufficient statistical power to detect potential interaction effects. Conversely, although the sample size for “*Daily work and life*” was sufficient to yield a statistically significant interaction effect, the relative sparsity of these statements compared to policy-related discourse suggests that these results should be interpreted with caution. Such cell-size imbalances may affect the stability of the model and limit the extent to which cultural differences can be generalized across all types of pandemic-related topics.

## Data Availability

The original contributions presented in the study are included in the article/supplementary material, further inquiries can be directed to the corresponding author.
